# Best Practices for High-Quality Anterior Segment Optical Coherence Tomography Imaging of Eyes with the Port Delivery Platform Implant

**DOI:** 10.3390/diagnostics15243098

**Published:** 2025-12-05

**Authors:** Andres Emanuelli, Matthew Ohr, Joel Castro, Rick Laoprasert, Alisa Prager, Paul Latkany, Glenn J. Jaffe

**Affiliations:** 1 Retina Care, Emanuelli Research and Development, Arecibo 00612, Puerto Rico; 2 Department of Ophthalmology, School of Medicine, University of Puerto Rico, San Juan 00612, Puerto Rico; 3 Department of Ophthalmology and Visual Science, The Ohio State University, Columbus, OH 43210, USA; 4 Department of Microbiology, University of Puerto Rico, Arecibo 00612, Puerto Rico; 5 Department of Ophthalmology, Duke University, Durham, NC 27710, USA; 6 Genentech, Inc., South San Francisco, CA 94080, USA

**Keywords:** anterior segment, anterior segment optical coherence tomography, optical coherence tomography, Port Delivery Platform

## Abstract

**Background/Objectives:** Anterior segment optical coherence tomography (AS-OCT) is a non-invasive imaging modality used to evaluate anterior segment features. This report aims to inform clinicians of best practices to obtain high-resolution AS-OCT images of anterior segment features of eyes implanted with the Port Delivery Platform (PDP). **Methods:** In PDP trials, AS-OCT imaging of the anterior segment was performed using Heidelberg Spectralis OCT (Heidelberg Engineering GmbH, Heidelberg, Germany) equipped with the Anterior Segment Module. Images from over 2500 separate study visits were obtained using standardized imaging parameters. The following stepwise approach was recommended to properly orient the volume scans over the extrascleral flange of the PDP ensuring that the scans are centered on the implant with adequate depth: (1) the volume scan was aligned such that: (i) the long axis of the scan was oriented parallel to the implant flange long axis, (ii) the imaging field was centered on the implant septum center, and (iii) it covered the entirety of the implant, with equal margins on either side of the implant flange; (2) the depth of the scan focus was adjusted to ensure that the conjunctiva and Tenon’s capsule over the overmold, as well as sclera under the implant flange, and the septum of the implant, were captured; and (3) Steps 1 and 2 were then repeated after the scan orientation was changed so that the short-axis scans were oriented parallel to the implant flange short axis. **Results:** Utilization of AS-OCT during clinical development of the PDP allowed visualization of anterior segment features, including the conjunctiva, Tenon’s capsule, and sclera, surrounding the PDP. Overall, these best practices enabled detailed structural imaging of the implant’s interface with surrounding ocular tissues. Common errors resulting in poor AS-OCT image acquisition included off-center raster scans, scans not being aligned parallel to the long and/or short axes of the PDP implant, or not being oriented along the implant axes, and inappropriate scan depths. **Conclusions:** Application of a standardized AS-OCT imaging procedure was used to obtain high-quality, high-resolution images of anterior segment features in the presence of the PDP implant. The best practices reported are not a requirement for managing eyes with the PDP, but a recommendation for how to obtain high-quality images of the anterior segment of eyes with the PDP implant.

## 1. Introduction

Anterior segment (AS) optical coherence tomography (OCT) is an imaging modality used to evaluate AS features, including the cornea, conjunctiva, sclera, assessment of the iridocorneal angle, and trabecular meshwork [1] in clinical practice. Optimized procedures to acquire AS-OCT images are needed and are especially important for multicenter clinical trials to compare image data among clinical study sites and to ensure reproducibility of findings.

AS-OCT is a non-contact, non-invasive imaging modality that utilizes low-coherence interferometry to provide cross-sectional images of AS features [1]. AS-OCT is commonly used with two technologies: spectral-domain (SD) and swept-source (SS). SD and SS AS-OCT use a fixed reference mirror and Fourier transformation analysis, enabling rapid imaging speeds and improved axial resolution [2,3]. The longer wavelength and faster scanning speeds used in SS technology offer increased depth of visualization [4] compared with SD technology [4] (100,000 A-scans per s for SS-OCT vs. 26,000–70,000 A-scans per s for SD-OCT [3]). Increasing use of OCT as an important tool to diagnose and manage ocular conditions has since led to the development and commercialization of SD and SS AS-OCT devices, which offer improved image resolution. The Spectralis OCT (Heidelberg Engineering GmbH, Heidelberg, Germany), RTVue, iVue (Optovue, Inc., Fremont, CA, USA), and Cirrus^®^ 6000 (Carl Zeiss Meditec, Inc., Dublin, CA, USA) are SD-OCT devices that have both posterior and AS imaging capability via the use of an AS lens, thereby eliminating the need to purchase a separate AS-OCT device. SS-OCT devices include the Anterion^®^ SS-OCT (Heidelberg Engineering GmbH), which is a dedicated AS imaging device, and the Triton™ (Topcon, Tokyo, Japan), which has both posterior and anterior imaging capabilities.

The clinical potential of AS-OCT for detailed imaging and analysis of AS structures for the diagnosis and management of ocular conditions pre- and post-surgery has been reviewed [5,6]. The use of AS-OCT with implanted ophthalmic medical devices has also been reported. In 2010, Taban et al. utilized AS-OCT to evaluate scleral thickness following Retisert^®^ implantation [7]. Schipper et al. (2025) reported the application of AS-OCT to monitor the thickness of patch grafts following PAUL^®^ glaucoma implant surgery [8]. However, acquisition of high-quality high-resolution images in the presence of an ocular device presents technical challenges and considerations, including device interference, patient movement, anatomical variability, lack of automated image registration, and optimal resolution to visualize fine details around the implant.

The Port Delivery Platform (PDP) is a drug delivery system comprising a surgically implanted, refillable ocular implant, ancillary device, and associated drug product to provide continuous intraocular drug delivery [9] designed to alleviate the need for frequent intravitreal injections, thereby reducing the treatment burden for patients with retinal disease. The PDP with ranibizumab (PDS), an anti–vascular endothelial growth factor, is the first iteration of the PDP and approved by the U.S. Food and Drug Administration for neovascular age-related macular degeneration (nAMD), diabetic macular edema (DME), and diabetic retinopathy (DR) [10]. The PDS is surgically inserted under the conjunctiva at the pars plana with attention to the closure of the conjunctiva, and follow-up includes slit lamp evaluation of the overlying conjunctiva. AS-OCT was used as a complementary, optional, exploratory imaging modality to visualize the most external part (the flange) of the implant, which resides under the conjunctiva, during the clinical development of the PDS [11,12], and is neither a routine nor required imaging technique for the management of patients with the PDP implant. Here, we report a standardized procedure for optimal SD AS-OCT imaging of eyes with the PDP implant. There is currently no standardized procedure for AS-OCT imaging in eyes implanted with the PDP outside of clinical trials, highlighting a need for such guidance. This report aims to inform clinical best practices to obtain exploratory high-resolution Heidelberg Spectralis SD AS-OCT images of AS features in the presence of the PDP implant and is derived from experience during clinical development of the PDS and expert consensus.

## 2. Materials and Methods

Spectralis (Heidelberg Engineering GmbH) and RTVue, iVue (Optovue, Inc.) were the imaging platforms that were most frequently used by the study sites during clinical development of the PDS. In this report, we will detail the procedure used to acquire AS-OCT images using the Heidelberg Spectralis device, as it is commonly used by retina surgeons.

### 2.1. Imaging Methods

During clinical development of the PDS, exploratory AS-OCT images of the implanted PDP were acquired with the Heidelberg Spectralis SD-OCT system equipped with the manually exchangeable Anterior Segment Module (AS lens with Spectralis software version 5.7 or higher). After positioning the patient’s head on the chinrest of the machine, the patient’s fixation was directed downward and toward the nose to expose the PDP implant in the supertemporal quadrant using an external fixation light. The photographer raised the eyelid, if needed, with or without the help of an assistant, to better expose the superotemporal quadrant where the implant flange is located. To obtain the images, the following steps were performed:

(1) The 30° standard lens was first installed, and the focus was set to 20 diopters. The 30° lens was then exchanged for the AS lens, and the position number was set to zero.
(2) The following standardized imaging parameters were then applied in the software: The IR + OCT mode was activated, and the scan mode was set to “sclera” and preset “large”.
(3) Two volume scans, each with 41 B-scans, were acquired with a field-of-view of 15° (horizontal) × 10° (vertical), automatic real-time (ART) of 15, high resolution, and enhanced depth imaging (EDI) mode. One volume raster scan was oriented along the long axis of the implant flange, and one was oriented along the short axis of the implant flange.

To properly orient the volume scans over the flange of the implant, the following stepwise approach was used:

(1) The volume scan was aligned such that: (i) the long axis of the scan was oriented parallel to the implant flange long axis, (ii) the imaging field was centered on the implant septum center, and (iii) it covered the entirety of the implant, with equal margins on either side of the implant flange.
(2) The depth of the scan focus was adjusted to ensure that the conjunctiva and Tenon’s capsule over the overmold, as well as sclera under the implant flange, and the septum of the implant, were captured. To adjust the scan focus, the camera was moved slowly toward the eye until the near-infrared image was illuminated evenly, and the OCT was displayed within the lower third of the imaging window. The ART value was increased as necessary to achieve an adequate signal-to-noise ratio.
(3) Steps 1 and 2 were then repeated after the scan orientation was changed so that the short-axis scans were oriented parallel to the implant flange short axis.

### 2.2. Image Analysis

Although no formal AS-OCT analysis was conducted for the PDS clinical trials, AS-OCT images have been obtained in over 2500 separate study visits. Images may be analyzed to evaluate the position of the PDP implant relative to other AS landmarks, such as the conjunctiva, Tenon’s capsule, and sclera. The following parameters can be assessed: implant flange visibility, septum positioning, tissue interfaces, and scan alignment.

### 2.3. Quality Control Measures

The quality of AS-OCT images can be hampered by the presence of artifacts, rendering image interpretation difficult and potentially leading to incorrect diagnosis and inappropriate disease management. Artifacts arising in SD-OCT images are the result of software errors (e.g., mirror artifact), operator-related errors (off-center artifact), and/or patient-related errors (e.g., motion artifact) [13]. Given that OCT artifacts have the potential to affect quantitative and qualitative assessment of the PDP and surrounding tissues in the clinical and clinical trial setting [13], it is imperative to adhere to a standard procedure to reduce artifacts, thereby ensuring quality and interpretability of the image. AS-OCT scan settings help optimize the number of B-scans (ART settings) obtained and identify areas in relation to the implant. During clinical development of the PDP, different ART settings, varying numbers of B-scans, and different image acquisition areas were used to reduce artifacts and optimize image quality during AS-OCT scanning. To determine if the PDP implant was imaged per the acquisition procedure, the following considerations were made: (1) whether the scan was centered on the implant; (2) whether the scan depth was appropriate (i.e., sufficient to visualize the relevant structures); (3) whether a sufficient scan length and number of scans collected; and (4) whether the focus of the scan appropriate?

## 3. Results

A comparison of key OCT variables for anterior and posterior segment examination is shown in Table 1. For optimal AS-OCT image acquisition, patients do not look directly into the camera fixation light. Rather, the photographer identifies the appropriate fixation for optimal exposure of the PDP implant, and short- and long-axis imaging for width and depth sample analysis, respectively (Table 1).

**Figure 1:**
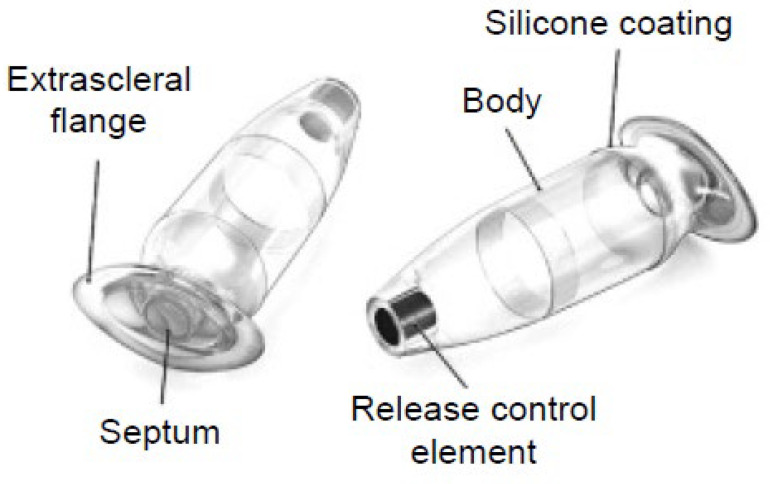
Schematic of the Port Delivery Platform (PDP). Reproduced with permission from Giulio Barteselli (on behalf of the authors), The Port Delivery System with Ranibizumab for Neovascular Age-Related Macular Degeneration [9].

**Figure 2:**
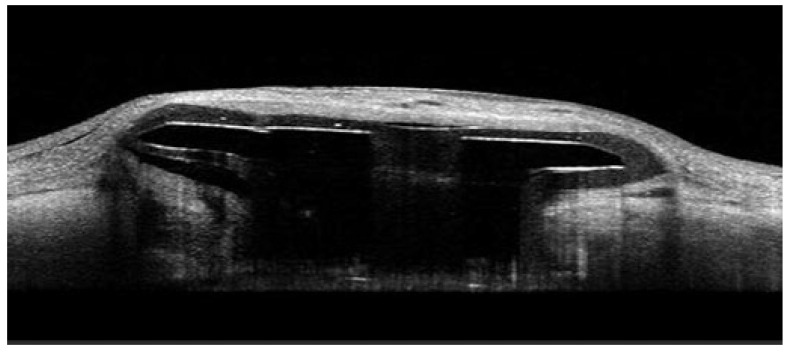
Anterior segment optical coherence tomography image of an eye with the Port Delivery Platform (PDP) implant. Image shows cross-section of the implant flange up to the neck of the PDP implant.

A schematic representation of the PDP is shown in Figure 1, which shows PDP components, including the septum and extrascleral flange [9]. An example AS-OCT image of the PDP embedded in the sclera is shown in Figure 2.

**Figure 3:**
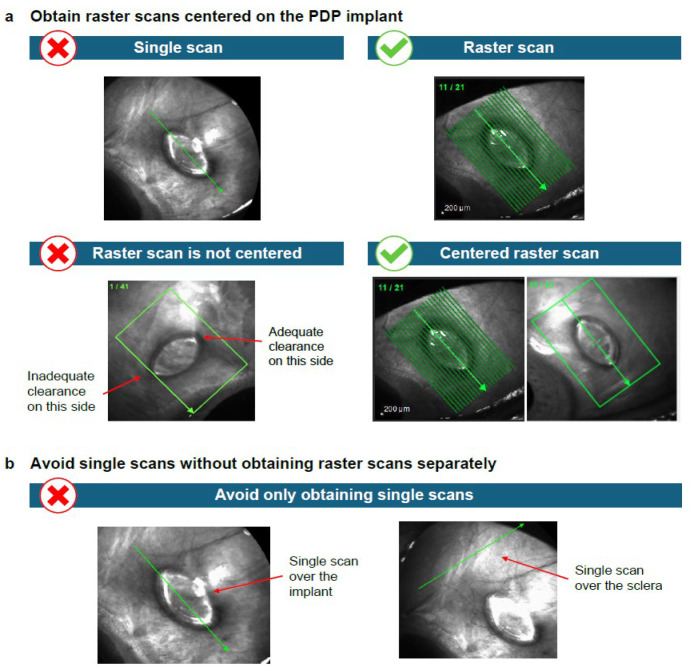
Anterior segment optical coherence tomography imaging in the presence of the PDP: Obtaining centered raster scans. Raster scans can be indicated by either multiple lines (**left**) or a box (**right**), depending on the number of scans obtained. Green lines denote individual B-scans and green arrows denote the B-scan direction. Abbreviations: PDP = Port Delivery Platform.

**Figure 4:**
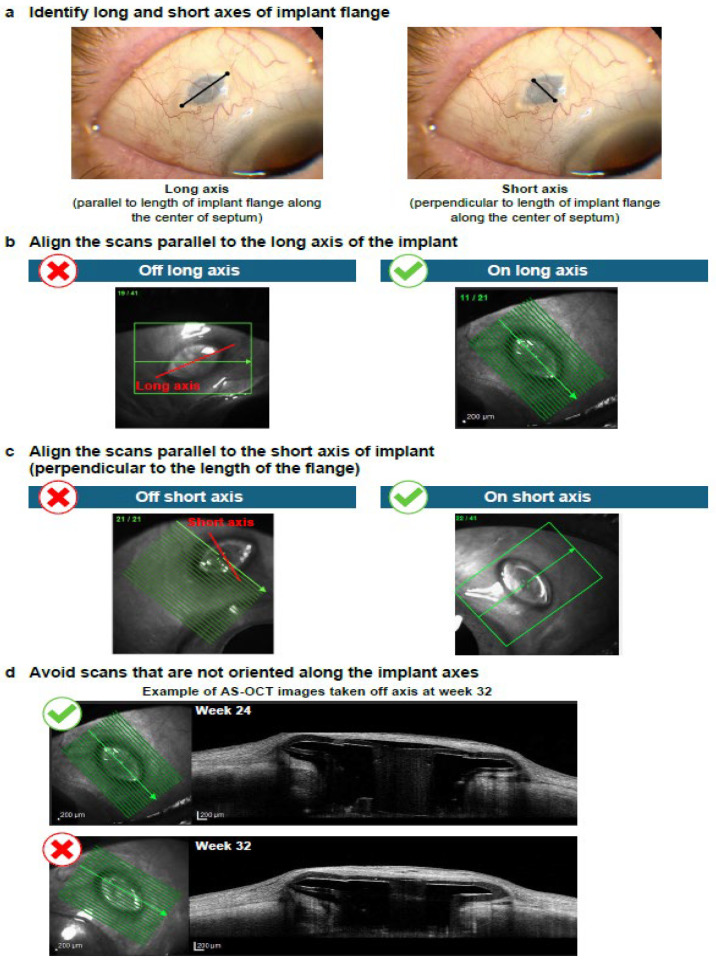
Anterior segment optical coherence tomography imaging of an eye implanted with the Port Delivery Platform: Aligning scan along the long and short axes of the implant flange. Green lines denote individual B-scans and green arrows denote the B-scan direction.

**Figure 5:**
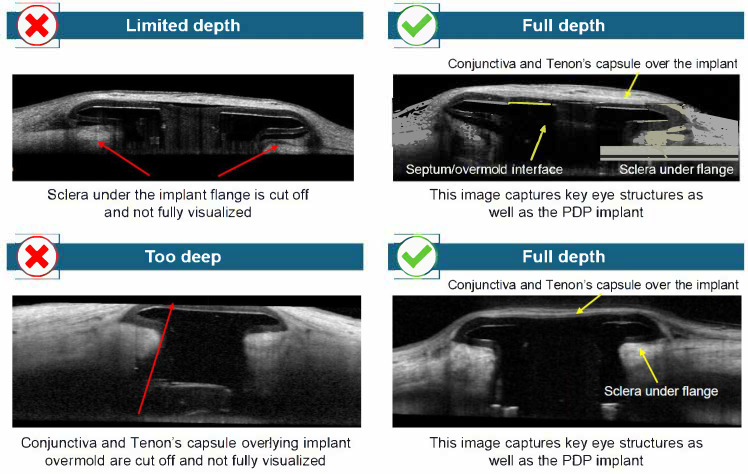
Anterior segment optical coherence tomography imaging in the presence of the PDP: Obtaining adequate depth of the scan. Abbreviations: PDP = Port Delivery Platform.

AS-OCT images demonstrating key technical aspects required for optimal imaging of the AS when the PDP is implanted are shown in Figure 3, Figure 4 and Figure 5. For optimal imaging, raster scans centered on the implant should be obtained (Figure 3). Raster scans should capture the entirety of the PDP implant, including temporal and nasal flange edges and adjacent sclera; however, it is important to note that complete visualization of the sclera beneath the implant flange may not be possible. Following identification of the long and short axes of the PDP implant flange (Figure 4a), the scans should be aligned parallel to the long axis and then the short axis of the PDP implant flange (Figure 4b and Figure 4c, respectively). Scans that are not oriented along the implant flange axes should be avoided (Figure 4d). Finally, the entirety of the PDP and relevant AS features should be captured (Figure 5).

## 4. Discussion

By developing and reporting a standardized procedure for AS-OCT acquisition in eyes with the PDP implant, we have provided ophthalmologists with parameters to aid the acquisition of consistent, high-quality, high-resolution images for the PDP. The best practices reported are not a requirement for managing eyes with the PDP, but a recommendation for how to obtain high-quality images of the AS in eyes with the PDP implant.

Utilization of AS-OCT during clinical development of the PDP allowed visualization of AS features, including the conjunctiva, Tenon’s capsule, and sclera surrounding the implant, and may potentially be used to supplement slit-lamp examination. The implant flange and septum were generally well defined in both long- and short-axis scans, particularly when raster scans were centered and aligned with the axis of the device. The conjunctival and Tenon’s capsule layers could be distinguished in most cases, and there was variable overlying tissue thickness among eyes with the PDP implanted. The scleral interface beneath the implant was visualized with adequate depth in EDI mode, allowing for assessment of implant depth and any potential tissue reaction or shadowing. Overall, the procedure enabled detailed structural imaging of the implant’s interface with surrounding ocular tissues. Techniques that consistently improved image quality included external fixation positioning and eyelid adjustment. Common errors resulting in poor image acquisition included raster scans that were not centered, leading to inadequate clearance on one side of the PDP implant flange; scans that were not aligned parallel to the long and/or short axes of the PDP implant flange; and scans obtained at inappropriate scan depths. Inappropriate scan depth resulted in ocular features not being fully visualized in AS-OCT images (e.g., full visualization of the conjunctiva and Tenon’s capsule was not achieved when the scan depth was too high). No overall differences in image quality or anatomical detail were observed between long- and short-axis scans. The orientation of the scans, whether on the long axis or short axis, did not impact image detail quality.

In addition to the general limitations of AS-OCT, there are several technical challenges that can lead to poor image quality that can be overcome by adhering to a standardized protocol. These technical challenges include the presence of artifacts (motion, shadow), anatomical variations, and consistent/comparable image acquisition and analysis procedures. Furthermore, the PDP can interfere with the visualization of the fine details surrounding the implant. To overcome these technical challenges, we propose the following best practices for optimal AS-OCT image acquisition of AS features in the presence of the PDP implant: (1) obtain raster scans; (2) align scans parallel to the PDP implant; (3) confirm adequate depth of image and capture AS features of interest; and (4) increase signal averaging by increasing ART, if needed. Techniques that consistently improve image quality, such as external fixation positioning or eyelid adjustment, are also encouraged, as the photographer has a more active role in obtaining optimal fixation through the provision of active feedback. In addition, proper working distance and alignment are important because improper working distance and misalignment in OCT can lead to suboptimal images and incorrect interpretations of scans. These best practices are important for the following reasons. Raster scans capture multiple cross-sectional images of the PDP implant and surrounding ocular anatomy, showing features in their entirety and, therefore, allowing for more detailed analyses than a single line scan. Avoidance of scans not oriented along the PDP implant axes will ensure consistent capture of images on each axis, enabling analysis of the same AS features in the presence of the PDP across time points. Finally, visualizing the conjunctiva and Tenon’s capsule over the PDP implant aids the exploratory analysis of the PDP implant and surrounding structures.

Application of a standardized AS-OCT imaging procedure for eyes implanted with the PDP has the potential to inform future clinical trials, and its use may be explored in the clinical care of patients with nAMD, DME, or DR treated with the PDS for post-operative evaluation and safety monitoring. For example, Ericksen et al. (2022) showed that AS-OCT imaging could be used to monitor eyes implanted with the PDS and help identify potential complications such as conjunctival retraction and erosion [14]. In this study, AS-OCT images of six eyes implanted with the PDS from the phase 3 Archway trial of patients with nAMD showed an initial qualitative decrease in thickness of the overlying conjunctiva and Tenon capsule from 1 day to 4 weeks post PDS implantation, followed by minimal thinning through the remaining mean of 91 weeks and 3 days of follow-up [14]. AS-OCT imaging throughout the entire follow-up period helped the authors postulate that tissue edema leading to an artifactual increase in thickness at day 1 was the likely cause of this initial observation, which had resolved by week 4 [14]. A standardized AS-OCT imaging procedure for eyes implanted with the PDP is a step toward ensuring consistent and reproducible findings in the research and clinical practice settings.

There are limitations to our proposed standardized procedure for AS-OCT imaging of the PDP implant. This article focuses on the Heidelberg Spectralis OCT device (Heidelberg Engineering GmbH), which was used during the initial clinical development of the PDP because the Spectralis system is the one used most commonly by retinal surgeons and can acquire high-quality PDP images. However, other AS-OCT devices are available, and ongoing clinical studies of the PDP include the use of the RTVue, iVue (Optovue, Inc.). These studies will allow us to increase our knowledge of best practices and standardized procedures for different imaging devices, and, when combined with potential standardized image formats, may ultimately inform the standard of care [15]. In addition, AS-OCT image acquisition is an exploratory endpoint for the clinical development of the PDP. Because AS-OCT does not have automated image registration, AS-OCT image acquisition is a manual process that relies on the photographer to capture the image at the intended anatomical region at each time point. Therefore, there are ongoing technical challenges, such as the ability to visualize AS features cross-sectionally as well as longitudinally. Our proposed guidelines aim to address these issues. Finally, although the acquisition of intraoperative AS-OCT images is not discussed here, it has the potential to aid in imaging during surgery [16].

## 5. Conclusions

In conclusion, our findings show that application of a standardized acquisition procedure for AS-OCT imaging can be used to obtain high-quality, high-resolution images of AS features in eyes with the PDP implant. A standardized methodology will lead to increased comparability and reproducibility of research findings.

## Figures and Tables

**Table 1:** OCT variables for anterior segment and posterior segment examination.

Variable	Anterior Segment OCT	Posterior Segment OCT
Patient looks directly into camera fixation light	No	Yes
Photographer identifies the appropriate fixation for optimal imaging	Yes	No
Short- and long-axis imaging ^a^	Yes	No

Abbreviations: OCT = optical coherence tomography. ^a^ Centered on implant.

## Data Availability

For eligible studies, qualified researchers may request access to individual patient-level clinical data through a data request platform. At the time of writing the request platform is Vivli. https://vivli.org/ourmember/roche/ (accessed on 29 September 2025). For up-to-date details on Roche’s Global Policy on the Sharing of Clinical Information and how to request access to related clinical study documents, see here: https://go.roche.com/data_sharing (accessed on 29 September 2025). Anonymized records for individual patients across more than one data source external to Roche cannot, and should not, be linked due to a potential increase in risk of patient re-identification.

## Abbreviations

The following abbreviations are used in this manuscript:

ART	Automatic real-time
AS	Anterior segment
EDI	Enhanced depth imaging
OCT	Optical coherence tomography
PDP	Port Delivery Platform
PDS	Port Delivery Platform with ranibizumab
SD	Spectral domain
SS	Swept source
